# Imaging manifestations of unusual side effects of new anticancer medications

**DOI:** 10.1186/1470-7330-14-S1-P37

**Published:** 2014-10-09

**Authors:** B Parameswaran, K Moodie

**Affiliations:** 1 Peter MacCallum Cancer Centre, Melbourne, Australia

## Aim

To illustrate imaging manifestations of unusual side effects of some new anti-cancer medications.

Many of the new anti-cancer medications target specific biologic pathways and are expected to cause less adverse effects on healthy tissues. Some of these agents are associated with unusual side effects, recognition of which is clinically important.

Ipilimumab is a monoclonal antibody against cytotoxic T lymphocyte-associated antigen (CTLA)-4 used for the treatment of metastatic melanoma. Though it has been shown to increase overall survival rates, it also results in various immune-related adverse effects, one of which is hypophysitis.

Crizotinib is an anaplastic lymphoma kinase (ALK) and c-ros oncogene 1 inhibitor, approved for treatment of non-small cell lung carcinoma. Its side effects include the unusual formation of complex renal cysts which mimic renal abscesses or malignancy.

In this presentation, we illustrate the imaging manifestations of Ipilimumab associated hypophysitis, early recognition of which is important as cessation of the drug in affected patients leads to resolution of hypophysitis, and also demonstrate complex renal cysts associated with the use of Crizotinib, knowledge of which is important to avoid misdiagnosis of renal abscess/ malignancy and consequent inappropriate treatment or stoppage of Crizotinib.

